# Books Reviewed

**Published:** 1867-09

**Authors:** 


### Books Reviewed.

The Principles and Practice of Disinfection. By Robert Bartholow, A. M., M. D., Professor of Materia Medica and Therapeutics in the Medical College of Ohio, etc. Cincinnati: R. W. Carroll & Co., 1867.

Dr. Bartholow has given us a more careful digest of the principles and practice of disinfection than we have heretofore possessed. The views embodied in this monograph are based upon experimental observations; the investigations made to determine the toxic effects of gases resulting from putrifaction, being especially careful. Putrifactive decomposition, the author ascribes to the agency of diseased invisible germs or *materies morbi*, suspended in the atmosphere, the destruction of which becomes the office of disinfectants.

The classification of disinfectants adopted is that suggested by Dr. Herbert Baker, who divided them into three classes: "class 1, agents that chemically destroy the noxious compounds; class 2, agents that arrest chemical change; class 3, agents that physically restrain the noxious compounds." To heat, is assigned the foremost rank in the list of disinfectants, as possessing a larger range of adaptability than any other agent, possessing oxidizing powers, thus destroying noxious compounds, being anticeptic through descication and acting mechanically by causing atmospheric currents. Ozone from its oxidizing properties, he especially recommends as a de-oderizer and purifier of the air of the sick chamber, ranking iodine and sulphurous acid next; should, however, a more decided disinfection be required, than can be obtained from any one of these agents singly, the combination of all three is recommended.

The disinfection of water from disease producing products the author effects best, either by boiling or by filtering through a mixture of freshly-burned charcoal and sand, while that of clothing and bedding is most effectually accomplished by heat. The subject of public disinfection is very carefully reviewed and many valuable suggestions are made as to the best, cheapest and most reliable manner to destroy the deleterious eminations arising from any cause.

Notes on the Origin, Nature, Prevention and Treatment of Asiatic Cholera. By John C. Peters, M. D., Second Edition, with an appendix, New York: D. Van Nostrand, 192 Broadway, 1867.

Among the numerous manuals of late, published upon Asiatic Cholera, this work especially commends itself, for the thorough and systematic manner in which the Origin, Nature, Prevention and Treatment of Cholera is discussed. The views entertained upon the most interesting feature of the disease, viz: its propogation, the author claims to have been fully corroborated, by the experience, gathered from our late visitation. But little importance is attached by him to the influences exerted by winds on the distribution of the disease, and while their occasional influence under favorable circumstances is admitted, he considers the communications of individuals from infected districts, the contamination by the choleraic poison, of drinking water, and of clothes, to be the most potent agents in the distribution of the Epidemic, while no amount of filth, and diarrhœa can be productive of true Asiatic Cholera in the temperate zone without the addition of the peculiar cause, which eminate from the discharges of Cholera patients by vomiting and purging.

In the consideration of the treatment Dr. Peters has rather presented a *resumé* of remedies at the command of the practitioner in combating the disease, than suggesting any particular plan to be adopted, and admits that many cases recover under all kinds of treatment, proper hygienic conditions being procured. The claims of homœopathy, in having achieved a greater proportion of recoveries by *their system* are utterly refuted by the report of their own members put in charge of Cholera wards, to test the efficacy of homœopathic treatment, the conclusions arrived at by men having the largest hospital experience, as Fleishman, and others are, that "homœopathy fails completely," the recoveries in their best appointed wards being but five per cent. better than that of the Cholera Ship.

---

Ununited Fractures Successfully Treated, with remarks on the Operation. By Henry I. Bigelow, M. D., Professor of Surgery in the Medical College of the University of Harvard: David Clapp & Son, 1867.

This paper presents the history of eleven consecutive cases of ununited fractures, all but one (in which there existed interstitial absorption,) being successfully treated by the following operative procedure.] [A free incision should be made over that point of the false joint, where the bone approaches nearest to the surface; the irregularly interlocked extremities of the bone carefully divided and the ends turned out by flexion of the false joint. The ends being exposed "a conical or other regular incision is to be made in the ragged callus which overlies the periosteum at its tip, which should be then seized by strong-toothed forceps, and efforts made to tear it out of the rugous inequalities of the formerly inflamed bone." The periosteum being carefully protected "half-an-inch of sound shaft with an iregular or conical extremity is to be excised," and the end brought in apposition, to be retained by silver or plated copper wire. For this purpose holes are drilled a half-an-inch from the extremities and through one wall only, the wires being inserted from without, inward, in one end, and from within, outward, in the other.

The good results obtained by Dr. Bigelow in the treatment of this class of fractures, commends more extensive trial.

On Railway and other Injuries of the Nervous System. By John Eric Erichson, Fellow of the Royal College of Surgeons, Professor of Surgery, and of Clinical Surgery in University College, etc., etc. Philadelphia: Henry C. Lea, 1867.

This book comprises a series of lectures delivered at the University Medical College in the spring of 1866, in which the author aimed "to describe certain forms of Injuries of the Nervous System that commonly result from Accidents on Railways," and which are included under the following titles: I, Introduction. II, Effects of Severe Blows on the Spine. III, On Concussion of the Spine from Slight Injury.—IV, Concussion of the Spine from General Shock, Twists and Wrenches of the Spine. V, Symptoms and Pathology of Concussion of the Spine, of Diagnosis, Prognosis and Treatment.

The importance of these questions cannot be over-estimated. Medical men have been too apt to overlook or treat lightly slight injuries resulting to the spine or brain, but which subsequently have developed themselves into lesions of the nervous system of the gravest character. Not unfrequently cases have come under our observation in which a patient having thus been injured, feels himself quite able to assist his fellow-sufferers, subsequently showing evidences of nervous lesions, incompatible with mental effort, and sometimes even with the continuance of life. We believe that this work of Dr. Erichson will exert a great influence upon the mode of treatment adopted in this class of injuries, and will especially tend to harmonize the heretofore conflicting testimony of the profession in Courts in which such cases may come up for consideration.

---

Code of Medical Ethics adopted by the American Medical Association, revised to date. New York: Wm. Wood & Co., 61 Walker street, 1867.

Although the conduct of medical gentlemen towards each other is usually marked for its courteous character, the adoption of a universal standard possesses many advantages. The revised Code of Medical Ethics defines the proper course of conduct upon the following topics: 1st. Of the duties of physicians to their patients, and of the obligations of patients to their physicians. 2d. Of the duties of physicians to each other, and the profession at large. 3d. Of the duties of the profession to the public, and of the obligations of the public to the profession.

